# Defining novel parameters for the optimal priming and expansion of minor histocompatibility antigen-specific T cells in culture

**DOI:** 10.1186/s12967-015-0495-z

**Published:** 2015-04-19

**Authors:** Valérie Janelle, Cédric Carli, Julie Taillefer, Julie Orio, Jean-Sébastien Delisle

**Affiliations:** Centre de recherche de l’Hôpital Maisonneuve-Rosemont, Montreal, Quebec Canada; Division of Hematology-Oncology, Hôpital Maisonneuve-Rosemont and Department of Medicine, University of Montréal, Montreal, Quebec Canada

**Keywords:** HA-1, Minor histocompatibility antigen, T cells, Exhaustion, Adoptive immunotherapy, Leukemia

## Abstract

**Background:**

Adoptive transfer of minor histocompatibility antigen (MiHA)-specific T cells is a promising therapy for patients with hematological cancers. However, the efficacy of the transferred cells is hampered by the acquisition of terminal effector differentiation and exhaustion features during expansion *in vitro* thus preventing their function and persistence *in vivo*. Yet, the factors that induce T-cell differentiation and functional impairment in culture remain poorly defined and are likely to vary depending on the method used for expansion.

**Methods:**

Using the clinically relevant HLA-A0201-restricted MiHA HA-1 as well as reagents and procedures that are readily transferable to a clinical environment, we designed a novel culture protocol and defined how exhaustion features appeared in function of time. The optimal time points for the expansion of “fit” MiHA-specific T cells were delineated using phenotypic and functional assessments including KLRG-1 and PD-1 surface markers as well as Ki67 staining and cytokine secretion assays.

**Results:**

Following a priming phase, an enrichment step and a rapid expansion stage, our method generates MiHA-specific T-cell lines. Evidence of phenotypic and functional dysfunction appear in function of culture duration, but display different characteristics following the extension of the priming or rapid expansion phases. While repeated antigen exposure during the priming phase induced the decline of the antigen-specific population and the expression of PD-1 and KLRG-1 on antigen-specific CD8^+^ T cells, the prolongation of an antigen-free expansion phase induced proliferation arrest and the relative loss of antigen-specific cells without impairing polyfunctional cytokine secretion or inducing PD-1 and KLRG-1 expression. A similar pattern was also observed after stimulating a virus-specific memory repertoire, except for the more rapid acquisition of exhaustion features upon repeated antigen exposure.

**Conclusion:**

Our results offer novel insights on the impact of culture duration on the acquisition of T-cell exhaustion features. Using a new clinical-compliant protocol, we define critical parameters to monitor in order to optimally differentiate and expand MiHA-specific T cells in culture prior to adoptive transfer.

**Electronic supplementary material:**

The online version of this article (doi:10.1186/s12967-015-0495-z) contains supplementary material, which is available to authorized users.

## Background

Allogenic hematopoietic stem cell transplantation (HSCT) can cure hematological malignancies refractory to cytotoxic therapy. The therapeutic potential of HSCT largely depends on the so-called graft-versus-leukemia (GVL) effect mediated by donor T cells recognizing alloantigens on the malignant cells [1-3]. In most cases, HSCT donors and recipients are fully matched at Human leukocyte antigen (HLA) loci, making Minor histocompatibility antigens (MiHA) the main targets of the GVL effect. MiHAs are naturally processed peptides from polymorphic endogenous proteins that are loaded onto HLA molecules and presented at the cell surface [4]. At this time, MiHA-based immunotherapy in the setting of HSCT is one of the most potent forms of cancer treatment, but it remains non-specific and can lead to widespread anti-host alloreactivity in the form of Graft-versus-host disease (GVHD) [5]. Hence, the differentiation, expansion, and adoptive transfer of hematopoietic-restricted MiHA-specific T cells is an attractive approach to augment the GVL effects without risking GVHD [6,7].

The adoptive transfer of MiHA-specific CD8^+^ T-cell clones and lines in humans has yielded mixed results [1,8]. Globally, some objective leukemia responses were seen, but no long-term evidence of leukemia control or cure were recorded. A likely explanation for the limited success of these pioneering studies was the lack of persistence of the T cells after transfer. Large scale *ex vivo* T-cell expansion and effector differentiation can lead to robust antigen-specific cytolysis *in vitro*, but also to terminal effector differentiation and poor capacity to further expand and persist *in vivo*. Accumulating evidence from both animal and human studies suggest that optimal therapeutic effects are achieved when the *ex vivo* generated T cells maintain features associated with early memory differentiation [9,10]. Hence, a compromise must be sought to ensure efficient antigen priming while limiting effector differentiation during the culture period.

We developed a clinical-compliant protocol based on procedures and reagents that have been used in clinical trials before or that are manufactured clinical grade to prime and expand T-cell lines against HA-1, a HLA-A0201-associated, immunodominant and hematopoietic-specific MiHA [11,12]. Our approach is based on a priming phase in gas-permeable culture vessels (G-Rex), where responder cells are cocultured with antigen-loaded dendritic cells, followed by an enrichment step and a rapid expansion protocol (REP). We aimed at identifying the optimal time points to obtain T-cell lines comprising functional and non-exhausted MiHA-responsive T cells. To this end, we evaluated the effects of culture duration at each step by assessing the expression of terminal differentiation markers and evaluating T-cell functionality. Our data support that phenotypic and functional exhaustion features were different according to culture stage (priming versus expansion) implying that the evaluation of T-cell fitness for immunotherapy must rely on several parameters that are greatly influenced by the type and duration of culture method. Hence, we propose a novel clinical-compliant protocol to generate and expand MiHA-specific T cells which takes these parameters into account.

## Methods

### Donors

Healthy volunteers expressing the HLA-A0201 allele had their HA-1 genotype determined by SBTexcellerator kit (GenDX, Utrecht, The Netherlands) and were selected on the basis of the HA-1^RR^ genotype (not endogenously expressing HA-1)[12]. Peripheral blood mononuclear cells (PBMCs) were obtained by venipuncture or apheresis followed by manual (Ficoll-Paque, GE Healthcare, Baie d’Urfe, QC) or automated (Sepax system, Biosafe America Inc., Houston, TX) gradient density separation. This study was approved by the local Research Ethics Committee. Epstein-Barr virus serological status was determined by detection of anti-Viral capsid antigen (VCA) IgG and Epstein-Barr nuclear antigen (EBNA) by immunofluorescence in our local clinical diagnostic laboratory.

### Dendritic cell (DC) generation

Monocytes from PBMCs were isolated by plastic adherence and cultured in DC medium (X-vivo 15, 5% human serum, 1X PSG, 1 mM sodium pyruvate) supplemented with 800 IU/mL GM-CSF (Feldan, Quebec, QC) and 1000 IU/mL IL-4 (Feldan). Dendritic cells were matured with GM-CSF, IL-4, TNFα (10 ng/mL), IL-1β (10 ng/mL), IL-6 (100 ng/mL) (Feldan) and prostaglandin E2 (1 μg/mL) (Sigma-Aldrich, Oakville, ON).

### T-cell line generation

Antigen-specific T cell lines were generated using 15 × 10^6^ PBMCs as responder cells and cocultured with autologous, peptide-loaded mature DCs as antigen presenting cells (APCs) at a 1:10 ratio (stimulator:effector). After 40 Gy irradiation, the DCs were loaded with 1 μg/mL HA-1 (VLHDDLLEA) (Genscript, Piscataway, NJ) or LMP2_426-434_ (CLGGLLTMV) (Anaspec, Fremont, CA). Cells were cocultured for 7 days in T-cell medium (Advanced RPMI 1640, 10% human serum, 1X L-glutamine) supplemented with IL-21 (30 ng/mL) and IL-12 (10 ng/mL) (Feldan) in a G-Rex10 vessel (Wilson Wolf Manufacturing, New Brighton, MN). At day 7, T cells were washed and restimulated with peptide-pulsed DCs and incubated in T-cell medium supplemented with IL-21, IL-2 (100 IU/mL), IL-7 (10 ng/mL) and IL-15 (5 ng/mL) (Feldan) for an additional week. Restimulations of T cells were performed weekly on day 14 and day 21 (up to 4 stimulations) in T-cell medium supplemented with IL-2, IL-7 and IL-15. Cytokines were replenished with half medium change at day 10, 18 and 25. The cell concentration was adjusted to 0.5 × 10^6^ cells/mL each week.

After 21 days, IFNγ-secreting cells from G-Rex culture were selected with the IFNγ Secretion Assay - Detection Kit (Miltenyi Biotec, San Diego, CA) according to the manufacturer’s instructions. Briefly, T cells were stimulated for 4 hours with appropriate antigenic peptide, labeled with an IFNγ catch reagent and an IFNγ detection antibody conjugated to R-phycoerythrin (PE) and magnetically harvested using anti-PE MicroBeads and a MACS separator (Miltenyi Biotec).

Selected IFNγ-secreting T cells were expanded *in vitro* using an adaptation of a previously described rapid expansion protocol (REP) [13]. Following IFNγ capture, approximately 5 × 10^4^ T cells were resuspended in 25 mL of T-cell medium containing 25 x 10^6^ irradiated (40 Gy) autologous PBMCs, 30 ng/mL OKT3 and 50 IU/mL IL-2 and transferred to a T25 tissue culture flask for 21 days. After 4 days, cultures were harvested and resuspended in 25 mL of fresh T-cell medium with 50 IU/mL IL-2. Half medium changes were performed every 3-4 days until the end of the culture. Cells were harvested at day 12 and 21 of the REP culture for analysis.

### IFNγ enzyme-linked immunospot assay (ELISpot)

ELISpot assays were used according to the manufacturer’s instructions (Mabtech Inc., Cincinnati, OH) with 1 × 10^5^ cells. Corresponding spot forming cells and activity per 1 × 10^5^ cells were determined on a vSpot Reader Spectrum (AID, Strassberg, Germany).

### Flow cytometry

#### Immunophenotyping

The phenotype of T cells was assessed at different time points of the culture by flow cytometry. To determine the antigenic specificity, HLA-A0201/HA-1 dextramer (Immudex, Copenhagen, Denmark) or HLA-A0201/LMP2 pentamer (ProImmune, Sarasota, FL) staining was performed. 7-AAD was used to attest for viability. Cells were surface stained with monoclonal antibodies to: CD3, CD4, CD8, CD45RO, CCR7, CD11c, CD19, CD56, CD14 (BD Biosciences, Mississauga, ON), CD62L, PD-1, CD57, KLRG-1 (BioLegend, San Diego, CA) or CD8 (eBiosciences, San Diego, CA), washed and fixed in PBS 2% FBS 1% PFA before acquisition on a LSRII instrument (BD Biosciences, Mississauga, ON). To determine the TCR repertoire diversity of antigen-specific CD8^+^ T cells, the analysis of Vβ distribution was performed using the IO Test Beta Mark TCR Vβ Repertoire kit (Beckman Coulter, Mississauga, ON) according to the manufacturer’s instructions on HLA-A0201/HA-1 dextramer positive cells. All data were subsequently analyzed using Flowlogic software (Inivai Technologies, Mentone, Australia).

#### Intracellular staining

To evaluate T-cells function, cells were incubated with 5 μg/mL antigenic peptides in the presence of brefeldin A (7.5 μg/mL) (Sigma-Aldrich), anti-human CD28/CD49d as costimulatory reagents (1 μg/mL) (BD Biosciences) for 4 hours. Subsequently, cells were stained for surface markers, then fixed, and permeabilized for intracellular staining using Foxp3 staining buffer set from eBiosciences according to the manufacturer’s instructions. Permeabilized cells were then incubated with IFNγ, IL-2, TNFα (BD Biosciences), Ki67 (BioLegend) antibodies (BD Biosciences) and resuspended in PBS 2% FBS 1% PFA before acquisition. For degranulation potential, BD GolgiStop solution, anti-CD107a and anti-granzyme B (BD Biosciences) were used. T cells treated with phorbol 12-myristate 13-acetate (PMA; 50 ng/ml) and ionomycin (500 ng/ml) (Sigma-Aldrich) were used as positive controls. Acquisition was performed with a LSRII flow cytometer and data were analyzed using Flowlogic software.

### Cytotoxicity assay

Specific cytotoxicity of T cells was measured in a standard 4 hour Cr^51^ release assay using autologous or allogenic Phytohemaglutinnin (PHA, Sigma-Aldrich) blasts labeled with Cr^51^ and loaded or not with antigenic peptides. The percentage of specific lysis was calculated as followed: specific lysis = [(experimental release-spontaneous release)/(maximum release-spontaneous release)] × 100 [14] at a ratio of 40:1 (effector:target).

### Statistical analysis

Statistical analyses were performed with IBM SPSS Statistics 21 software (Armonk, NY). A Shapiro-Wilk Test of Normality showed that we could not always assume normally distributed data. Thus, a one-tailed Wilcoxon signed rank test was used for all comparisons. P values of less than 0.05 were considered significant.

## Results

### Antigen-presenting cells efficiently prime MiHA-specific T cells in G-Rex bioreactors

Based on established methods [1,8,15-17], we assessed the effect of repetitive weekly stimulation on the priming and expansion of MiHA-specific CD8^+^ T cells. We performed HA-1-loaded autologous dendritic cell (DC) stimulations of peripheral blood mononuclear cells (PBMCs) as a source of responder HA-1-specific CD8^+^ T cells for four different donors. This coculture was done in G-Rex bioreactors, as used by others [18], to maximize cellular expansion starting from only 15 million PBMCs. During this time, exogenous cytokines were introduced at different stages of the culture (IL-12 and IL-21 the first week; IL-21, IL-2, IL-7 and IL-15 the second week and IL-2, IL-7 and IL-15 for the subsequent weeks) to promote T cell differentiation, survival and expansion [19-23].

**Figure 1:**
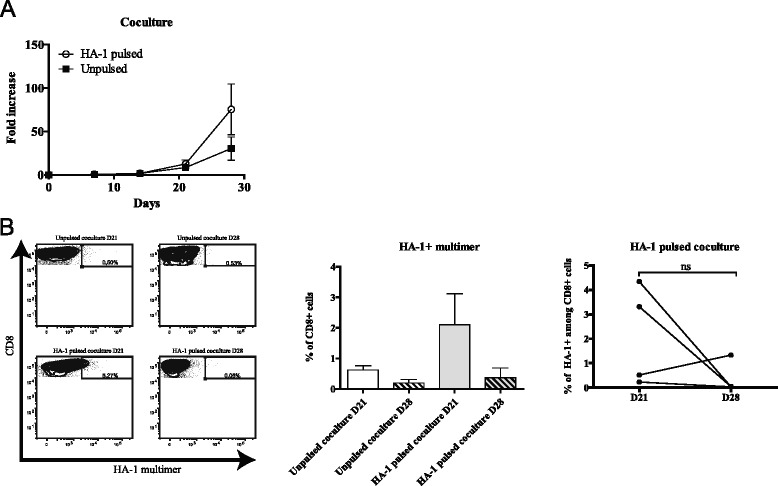
Generation of MiHA-specific T-cell lines in coculture. **(A)** Kinetics of culture growth as represented by the fold increase in cell count from the number of PBMCs seeded at the beginning of the coculture step; *n* = 4. **(B)** left: one representative of the CD8^+^ HA-1^+^ cells generated over time analyzed by flow cytometry; middle: combination of 4 donors, data show average of donors ± SEM; right: variation of CD8^+^ HA-1^+^ over time for each donor. ns (non significant).

We monitored this coculture for a total of four weeks. According to our conditions, T-cell lines entered their exponential growth phase around day 14 and reached their maximal growth rate between days 21 and 28 to achieve a total of ~25 to 140-fold expansion (Figure 1A). In parallel, cocultures with unpulsed DCs were performed as control and showed more modest expansions, although the difference was not statistically significant.

Since total PBMCs were used as the source of responder cells, we monitored for the presence of non-T cells and the subset distribution amongst CD3^+^ cells. We observed a balanced CD8/CD4 ratio (Additional file 1: Figure S1A). More than 90% of cells were CD3^+^ at each analyzed time points and we found that there was a negligible amount of B cells or monocytes/macrophages and a low but stable percentage of NK cells persisting over time (~5%) (Additional file 1: Figure S1B).

The proportion of multimer-positive cells varied extensively throughout the culture (Figure 1B and Additional file 2: Table S1) and displayed an oligoclonal Vβ chain expression pattern (Additional file 1: Figure S1C). The presence of HA-1 multimer-positive CD8^+^ T cells was found in all cell lines by day 21 but tended to decrease during the subsequent week (respective means of 2.1% to 0.36%). This implies that a minimum of three DC stimulations are needed to reliably achieve the generation of detectable MiHA-specific T cells and that prolongation of culture duration rapidly leads to a decline in the proportion of antigen-specific T cells.

### Longer coculture duration induces expression of markers indicative of T-cell terminal differentiation

**Figure 2:**
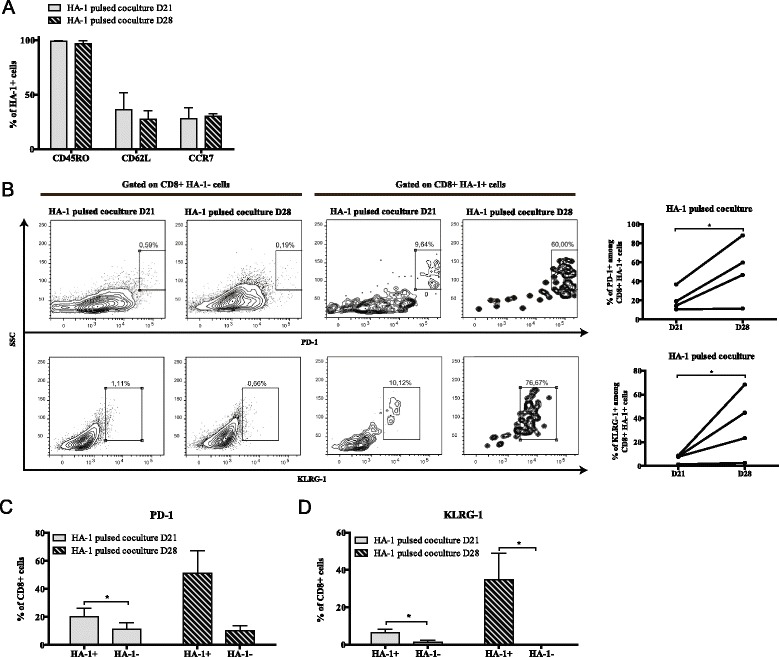
Induction of terminal differentiation marker expression in coculture over time. **(A)** CD45RO, CD62L and CCR7 expression on HA-1 multimer-positive cells. **(B)** left: representative plots and right: combination of PD-1 and KLRG-1 expression over time on HA-1^+^ and HA-1^-^ CD8^+^ T cells; *n* = 4. Comparison of **(C)** PD-1 and **(D)** KLRG-1 expression between the multimer-positive and negative CD8^+^ T-cell fractions within the cultures at different time points ; *n* = 4, data represented as average ± SEM. *, *P* < 0.05.

We then decided to compare cells harvested at 21 days (three DC stimulations) and 28 days (four DC stimulations). At both time point of the coculture phase, 30-40% of HA-1-specific CD8^+^ T cells displayed expression of CD45RO with CD62L and/or CCR7 (Figure 2A). This percentage did not change as a function of time suggesting that this system preserved the central memory phenotype typically associated with the early stages of T-cell differentiation and, in the context of adoptive immunotherapy, the capacity to further expand and persist *in vivo* [24]. Next, we characterized the differentiation status of HA-1-specific T cells by examining the expression of inhibitory receptors associated with T-cell dysfunction [25,26]. In cocultures, the proportion of HA-1-specific cells expressing PD-1 or KLRG-1 was higher at day 28 compared to day 21. The upregulation of these markers over time was also consistent among individual cell lines (Figure 2B). Thus, our 21 day coculture protocol enriched non-exhausted antigen-specific T cells while pursuing at day 28, using a fourth DC stimulation, increased PD-1 and KLRG-1 expression in a multimer-positive CD8^+^ T cell-specific manner (Figure 2C and D) hinting at the possibility that coculture-specific factors drive phenotypic exhaustion. Hence, our data suggest that time-dependent expression of terminal differentiation markers is distinctly regulated depending on the number of antigen-presenting cell stimulations.

### Cytokine capture followed by a rapid expansion protocol allows for enrichment of MiHA-specific CD8^+^ T cells

Knowing that four DC stimulations induced robust T-cell expansion but a relative decline and phenotypic exhaustion of antigen-specific cells, we focussed on day 21 cultures as a source of “fit” MiHA-responsive cells (i.e. optimal proportion of antigen-specific cells with low expression of markers showing evidence of terminal differentiation). In order to enrich for HA-1-specific cells, we opted for an IFNγ capture procedure at day 21 of the coculture. This procedure has the non-negligible advantage of having been used in clinical studies [27-30] and, as opposed to multimer-based sorting, it allows for the enrichment of IFNγ producing cells for different peptides or peptide libraries for which multimers may not be available. Following this step, cells were expanded with a rapid expansion protocol (REP) using anti-human CD3 antibody (OKT3) and IL-2 [13]. In order to address the main objective of this study, we monitored this culture from 12 to 21 days to define the impact of culture duration on T-cell phenotype and function.

**Figure 3:**
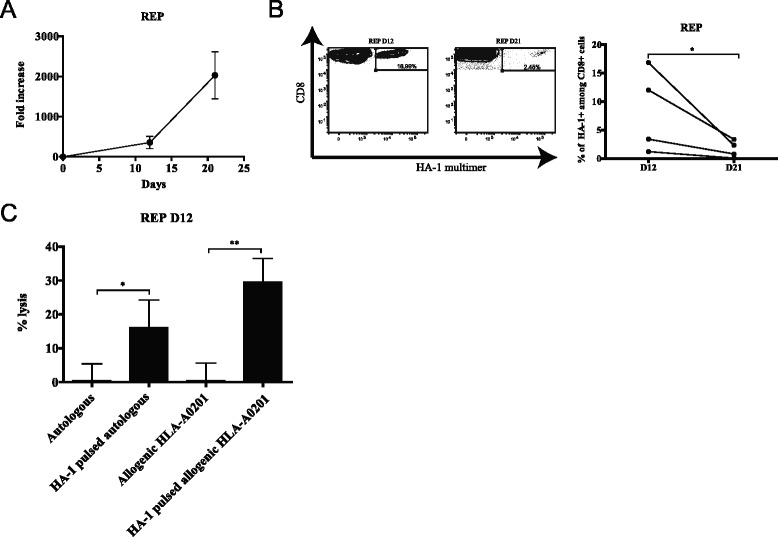
Expansion of MiHA-specific T-cell lines in REP. **(A)** Kinetics of culture growth as represented by the fold increase in cell count from the number of IFNγ-secreting cells seeded at the beginning of the REP step; *n* = 4. **(B)** left: one representative of the CD8^+^ HA-1^+^ cells generated over time analyzed by flow cytometry; right: variation of CD8^+^ HA-1^+^ over time for each donor. **(C)** Percentage of specific lysis measured with a Cr^51^ release assay of autologous or allogenic HLA-A0201 HA-1^Neg^ cells pulsed or not with HA-1 peptide; *n* = 4. *, *P* < 0.05; **, *P* < 0.01.

In the REP culture, cells rapidly expanded (~76 to ~640-fold) within the first 12 days and continued their growth to reach a maximum of ~2000-fold expansion after 21 days (Figure 3A). Similar to the initial coculture, the proportion of HA-1-specific cells fell between 12 and 21 days in REP in all cell lines (respective means of 8.36% to 1.64%) (Figure 3B).

T-cell lines primed *in vitro* contain large numbers of polyspecific T cells that are not specific for the targeted antigen(s). Nonetheless, clinical studies have shown that T-cell lines do not induce GVHD when injected into allogenic recipients [8,31]. In the perspective of clinical translation, the innocuity of the T-cell lines must be ascertained. We confirmed that after 12 days of REP, the cell lines were not cytotoxic to autologous or HLA-disparate, HA-1-negative targets (all sharing the HLA-A0201 allele but mismatched at 4 to 7/8 HLA loci) in the absence of the antigenic peptide. However, HA-1-dependent cytolysis could be demonstrated against both autologous and allogenic HLA-A0201 targets pulsed with peptide (Figure 3C). We also determined that T cells from the REP at day 21 remained cytotoxic when stimulated with HA-1 peptide but not with an irrelevant HLA-A0201-associated peptide as evidenced by CD107a and granzyme B co-expression (Additional file 3: Figure S2).

### Extending the REP induces proliferation inhibition without altering polyfunctionality

**Figure 4:**
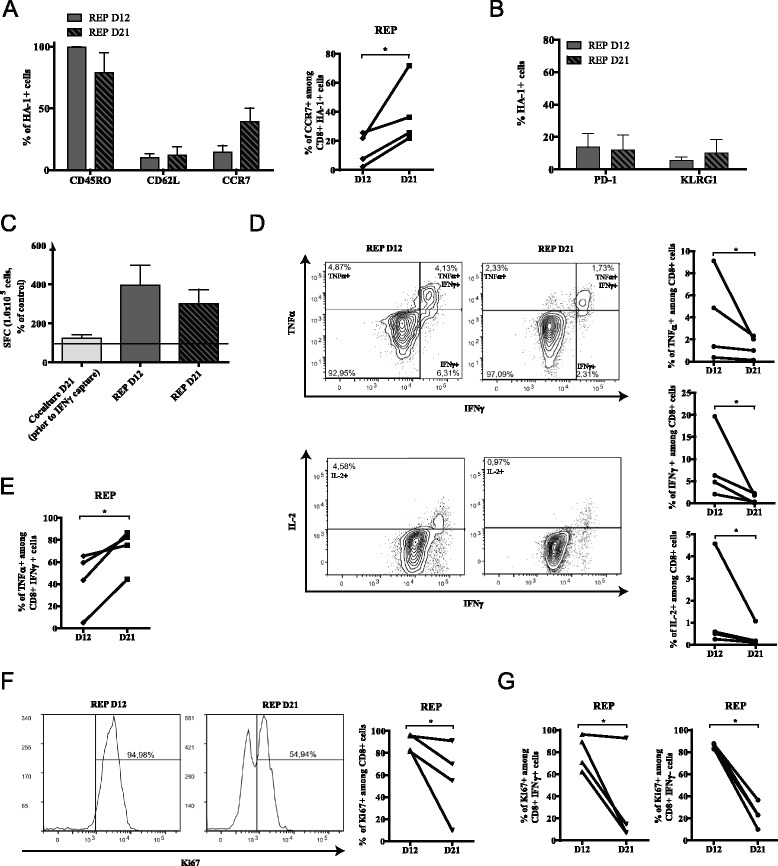
Induction of functional exhaustion in REP over time. **(A)** Left: CD45RO, CD62L and CCR7 expression on HA-1 multimer-positive cells in REP and right: increase of the percentage of CD8^+^ HA-1^+^ CCR7^+^ for each donor over time. **(B)** PD-1 and KLRG-1 expression over time on HA-1 multimer-specific CD8^+^ T cells; For A and B; *n* = 4, histograms represent means ± SEM. **(C)** Cells in each culture steps were harvested and stimulated *in vitro* with HA-1 peptide. Specific IFNγ spot forming cells (SFC) were measured and compared to non-stimulated cells with the ELISpot assay; dotted line represent detection threshold, *n* = 4, data show average ± SEM. **(D)** Cells from REP cultures were harvested over time and submitted to intracellular staining for flow cytometry analysis of TNFα, IFNγ and IL-2 production after HA-1 peptide restimulation; left: representative plots and right: representation in each culture. **(E)** Proportion of TNFα secretion in IFNγ-positive cells in every cell lines. **(F)** left: one representative and right: percentage of CD8^+^ Ki67^+^ for each donor in the REP over time. **(G)** comparison of Ki67 expression between the IFNγ-positive and negative CD8^+^ T-cell fractions within the cultures after HA-1 peptide restimulation. *, *P* < 0.05.

Comparable to what we have seen in the coculture phase, the proportion of HA-1 multimer-positive CD62L-expressing cells remained stable during the REP phase, but unexpectedly, the proportion of antigen-specific CD8^+^ T cells expressing CCR7 consistently rose at day 21 of the REP (Figure 4A). On the contrary, following IFNγ capture enrichment at day 21 of the coculture and further expansion in the REP, the proportion of PD-1 and KLRG-1 positive cells remained low (Figure 4B). Hence, the IFNγ capture procedure enriched non-exhausted antigen-specific T cells, and the REP culture allowed for their expansion without the induction of PD-1 or KLRG-1 expression.

We therefore sought to analyze, in more detail, the functionality of CD8^+^ T cells at the different time points of the REP culture. The IFNγ ELISpot assay provides a sensitive estimation of antigen-specific T-cell responses. In accordance with the frequency of HA-1 multimer-positive T cells found both at 12 and 21 days, we determined that the REP enhanced the proportion of IFNγ-secreting cells detected after re-exposure to HA-1 when compared to pre-IFNγ capture cocultures (Figure 4C). We further examined the antigen-specific response during the REP by intracellular flow cytometry to evaluate TNFα, IFNγ and IL-2 production by CD8^+^ T cells after peptide exposure *in vitro*. In a reproducible manner, the proportion of cytokine secreting cells declined over time in the REP (Figure 4D). Again, this correlated with the decreased percentage of multimer-positive cells present within the culture. Conversely, the proportion of polyfunctional T cells among the IFNγ-producing fraction increased over time (Figure 4E), suggesting that REP extension may favor the persistence or expansion of polyfunctional over monofunctional cells. However, the percentage of late REP CD8^+^ T cells (day 21) expressing Ki67 decreased indicating that the proportion of actively proliferating cells declined with time in REP (Figure 4F). Nevertheless, as demonstrated by intracellular staining following HA-1 peptide restimulation, the fall in actively proliferating cells was not antigen-dependent since HA-1-reactive IFNγ-secreting cells were similarly affected when compared to non-reactive IFNγ-negative cells (Figure 4G). Our results clearly indicate that REP duration adversely impact the proportion of antigen-reactive cells and globally blunts the proliferative response of antigen-reactive and non-reactive T cells.

### The kinetics of exhaustion vary depending on the nature of the antigen

**Figure 5:**
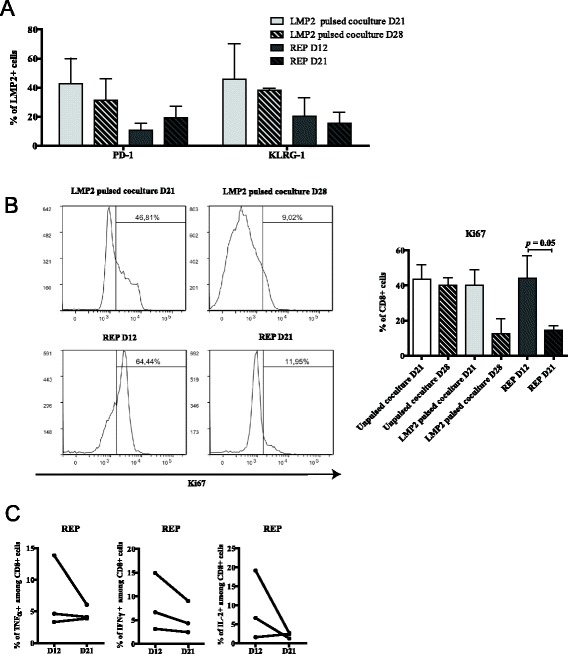
Kinetics of phenotypic and functional exhaustion profile depending on the nature of the stimulating antigen. **(A)** CD8^+^ LMP2_426-434_^+^ cells expressing PD-1 and KLRG-1 in both culture steps over time; *n* = 3, data represented as average ± SEM. **(B)** CD8^+^ T cells at each culture time points were analysed by flow cytometry for intracellular staining of Ki67; left: one representative and right: *n* = 3, data represented as average ± SEM. **(C)** Intracellular staining analysis of TNFα, IFNγ and IL-2 production after LMP2_426-434_ peptide restimulation at day 12 and 21 of the REP.

Since the development of exhaustion/dysfunction varies as a function of time and expansion method (coculture vs REP), it may also depend on the targeted antigen. We used 3 available donors from whom we had generated anti-HA-1 T-cell lines to assess whether similar or divergent features of the antigen-specific T cell phenotype would be observed following priming and expansion against another antigen. We selected a HLA-A0201-restricted peptide derived from the Epstein-Barr virus (EBV) antigenic protein LMP2. Viral antigens and MiHAs are both “foreign” peptide sequences capable of eliciting strong T-cell responses. A salient difference is that the 3 donors had all been previously exposed to EBV (positive serology - not shown). We could therefore evaluate whether our protocol would have a different impact on the stimulation of a memory instead of naïve repertoire in the same individuals. Following three peptide stimulations, LMP2_426-434_-specific CD8^+^ T cells represented up to 10% of CD8^+^ T cells following a total T-cell expansion comparable of that of HA-1 supplemented cultures (Additional file 4: Figure S3). However, the proportion of multimer-positive T cells expressing PD-1 or KLRG-1 was already high on day 21 of the coculture and remained stable on day 28 (Figure 5A). In stark contrast with our observation with HA-1, there was a substantial decline in the proportion of Ki67-expressing CD8^+^ T cells from day 21 to day 28 of the coculture (Figure 5B). This suggests that the acquisition of exhaustion features occurred more rapidly in the context of repeated exposure to the EBV-derived antigen. Likewise, Ki67 staining sharply fell from REP day 12 to REP day 21 with little variations in the proportion of antigen-specific CD8^+^ T cells expressing PD-1 or KLRG-1 (Figure 5A and B). Nonetheless, as for anti-HA-1 T-cell lines, antigen-specific cytokine secretion tended to decline between day 12 and day 21 of the REP in each independent sample tested (Figure 5C). Altogether, these results suggest that the acquisition of phenotypic and functional T-cell exhaustion features vary according to the targeted antigen in the same donors. This would imply that the timing of cell harvesting for adoptive transfer might need to be fine-tuned according to the targeted antigen.

## Discussion

In the context of HLA-identical hematopoietic cell transplantation, the anti-MiHA alloresponse is the basis of the GVL effect, one of the strongest forms of cancer immunotherapy. However, the adoptive transfer of *ex vivo* primed and expanded MiHA-specific T-cell clones or lines have failed to provide decisive anti-leukemia responses. With the caveat that previous anti-MiHA adoptive immunotherapy clinical studies were done in advanced, hard to treat leukemia patients, it is also possible that the *ex vivo* generated MiHA-specific T cells used in these trials had reached some level of exhaustion *in vitro* prior to adoptive transfer. In recent years, a growing consensus has emerged around the notion that the cytokine environment, repeated antigenic stimulations or culture duration used to generate a high number of antigenic-specific T cells can induce terminal T-cell effector differentiation and exhaustion thereby limiting *in vivo* therapeutic efficacy [15]. Determining the optimal culture conditions to generate less differentiated and non-exhausted T cells is thus highly relevant for adoptive immunotherapy. Moreover, defining the phenotypic or functional characteristics that reliably inform the prediction of antigen-specific T-cell fitness in culture or predict for *in vivo* efficacy is essential [32].

**Figure 6:**
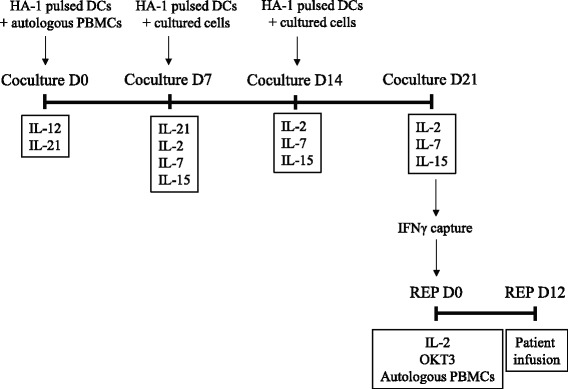
Proposed protocol for the priming, enrichment and rapid expansion of non-exhausted MiHA-stimulated T-cell lines. Schematic representation of the protocol showing the coculture and the REP with the associated cytokines used in cultures.

A paradigm shift is occurring in the field of adoptive immunotherapy after it was found in several models that therapeutic efficacy depended on the phenotype and the “early” differentiation of antigen-specific T cells rather than the number of cell infused. Hence a major focus of this work was to evaluate the impact of culture duration on T-cell differentiation and expansion. Based on these results, we propose a new protocol for the optimal generation of non-exhausted MiHA-specific T-cell lines (Figure 6). We have developed a strategy based on several previously described approaches. Our system is based on material and reagents that can be made clinical grade, and as such offers the advantage to be rapidly adaptable to a clinical environment. Moreover, it was designed to take advantage of a cytokine combination that has been shown to promote priming and enhance survival of antigen-specific T cells [22,23,33,34]. IL-15 is generally considered to be a regulator of T cell homeostasis because it works with other common γ-chain cytokines, like IL-2 and IL-7, to control the maintenance of naive and memory T cell populations as well as to promote the survival of antigen-specific CD8^+^ T cells. These latter cytokines were thus used from the second stimulation until the end of the coculture [20,35-38]. Since IL-21 has been shown to prevent apoptosis and terminal differentiation as well as further drive expansion of the cells responding to antigen stimulation when used in combination with IL-15 during the priming of antigen-specific CD8^+^ T cells, it was introduced early in the beginning of the coculture and kept for two weeks [19,39,40]. It may also program effector cells to reacquire central memory features after adoptive transfer and persist *in vivo* [19]. Finally, Il-12 was used only during the first week to take advantage of its capacity to promote naïve CD8^+^ T cell priming [41,42].

From only 15 million PBMCs as starting material, we aimed at maximizing DC-T cell interactions and cell growth while minimizing manipulations using a single gas-permeable vessel [17]. In order to enrich multimer-positive cells while limiting repeated stimulations, we decided to proceed to antigen-specific cell enrichment at day 21 of our coculture, which is comparable or shorter than previously published methods [1,6,32]. The IFNγ capture procedure for specific cells enrichment is amenable to several types of antigens and has been used in the past to enrich MiHA-specific cells [16]. This method is particularly useful when other methods of cell separation or enrichment, such as multimer-based sorting, cannot be done [43,27]. Enriched MiHA-specific cell lines were further expanded for 12 days according to an adaptation of a previously described REP procedure [13]. This has already been proven to rapidly and efficiently expand T cell clones or tumor-infiltrating lymphocytes (TIL), and, in our case, rapidly expanded cell lines enriched in HA-1-specific T cells [44,45].

Our protocol generates both central memory and effector memory phenotype T cells based on CCR7 and/or CD62L expression on CD45RO^+^ cells. According to previous studies, central memory T cells are predicted to have increased *in vivo* efficacy relative to effector memory T cells. However, there is also evidence that a fraction of effector memory T cells have the potential to revert back to a central memory phenotype when transferred into patient and persist, indicating that the acquisition of effector memory phenotype in culture may not always predict limited functionality *in vivo* [19,46]. Nevertheless, the proportion of effector memory or central memory phenotype cells in both culture conditions (coculture and REP) did not predict loss of antigen-specific cells or decline in their functionality. Thus, it was imperative to find complementary features that would better characterize specific T cell lines to attest their differentiation and functional status.

In the coculture step of our protocol, it is likely that repeated antigenic stimulation led to terminal differentiation, as evidenced by upregulation of PD-1 and KLRG-1 predominantly on antigen-specific cells. This corroborates previous results showing that repeated peptide-pulsed DCs exposure can adversely affect antigen-specific T-cell yield [15]. Thus, for the minor histocompatibility antigen HA-1 in our system, three DC stimulations (for a total of 21 days of culture) offered the best generation of specific T cells with a favorable differentiation status. This is further supported by the fact that the REP, where no specific antigen stimulation occurs, did not induce PD-1 and KLRG-1 expression over time.

In the REP, the decrease in the proportion of cytokine-producing cells was correlated with a loss of multimer-positive cells. However, the antigen-reactive cells persisting after 21 days of REP were polyfunctional, in greater proportion than at day 12. This was rather surprising as we expected that further IL-2 driven expansion would lead to terminal differentiation and functional impairment [47]. Given that the proportion of antigen-specific cells expressing CCR7, a central memory marker, is also increased after 21 days of REP, one can speculate that early differentiated, fit and polyfunctional T cells preferentially persisted in culture as compared to other antigen-reactive cells. However, it is also possible that IL-21 addition during the priming phase programmed the re-expression of memory markers at later REP times [15,19]. Nonetheless, the proportion of proliferating cells fell dramatically by 21 days in REP, suggesting the possibility that cells had acquired a certain degree of dysfunction. In the context where both antigen-reactive and non-reactive CD8^+^ T cells lost their proliferative response, one must consider that the relative decline in antigen-reactive cells might be due to increased apoptosis. Other studies have also reported that cell proliferation could be inhibited without altering the cytokine production of antigen-specific T cells [48]. In our REP procedure, cytokine production of MiHA-specific CD8^+^ T cells proved to be modulated by mechanisms divergent from proliferation as well. This could potentially be influenced by the production of nitric oxide (NO) synthase by autologous PBMCs within the culture which can have inhibitory effect on T-cell division without necessarily affecting cytokine secretion capacity [49,50]. Hence, in REP procedure, unlike in some other contexts [25,26], exhaustion appears to be independent of PD-1 and KLRG-1 expression or polyfunctionality but manifests itself by a drastic decline in proliferation and persistence and should therefore not last longer than 12 days. Thus, our protocol shows that an improved balance composed of no more than three DC stimulations followed by 12 days of antigen-specific IFNγ-secreting T-cell expansion would allow for the expansion of non-exhausted MiHA-specific T cells.

It was somewhat surprising that similar culture conditions led to different T-cell phenotypes when another antigen was used. As previously shown, the stimulation of a naïve repertoire requires more activation signals than the stimulation of memory T cells [51,52]. To compare the phenotype of *in vitro* generated MiHA-specific CD8^+^ T cells under different conditions to another peptide, we purposely chose an antigen that would stimulate a memory repertoire rather than a naïve repertoire in more than 90% of adults. LMP2_426-434_ –specific T cells, as opposed to HA-1 specific T cells, more rapidly expanded but displayed exhaustion marker expression, perhaps as a consequence of less stringent requirements for activation, a lower dependency on costimulatory signals and a shorter requirement of antigenic stimulation [53,54]. This may also be a matter of TCR affinity implying that a stronger interaction lead to a more robust T-cell response [55]. The functional avidity can also impact CD8^+^ T cell activation since it is considered as a quantitative determinant of the activation threshold of a T-cell clone [56,57]. Hence, differentiation and expansion protocols used for stimulating a memory repertoire might need to be optimized depending on affinity, avidity and progenitor frequency.

## Conclusion

Altogether our results indicate that phenotypic and functional exhaustion are greatly affected by duration of the culture and display different characteristics according to the culture conditions. Accordingly, we propose a novel clinical-compliant protocol taking these parameters into account to generate MiHA-specific CD8^+^ T cells of optimized quality. Since no single biomarker is sufficiently sensitive or specific on its own to adequately determine levels of T-cell differentiation and functionality, the simultaneous use of several markers such as PD-1, KLRG-1, IFNγ and Ki67 may help to predict *in vivo* persistence of cultured specific T cells. A better understanding of the mechanisms by which a given antigen or a specific priming/expansion system contribute to the induction of specific T-cell exhaustion will be extremely valuable for the *ex vivo* generation of healthy T cells for clinical applications.

## Additional files

Additional file 1: Figure S1. Cell subpopulations and characteristics throughout cultures steps. **(A)** CD4^+^ and CD8^+^ T cells proportions analyzed by flow cytometry; *n* = 4, data show average ± SEM. **(B)** Percentage of contaminant such as B cells, NK cells and monocytes/macrophages analyzed by flow cytometry; *n* = 4, data represented as average ± SEM. **(C)** Clonogram representation (with representative examples in top right corner) of formerly identified TCR Vβ repertoire in CD8^+^ HA-1^+^ (upper panel) and CD8^+^ HA-1^-^ (lower panel) cells at day 12 of the REP; *n* = 3, averages ± SEM. Additional file 2: Table S1. Number of total and HA-1-specific cells generated over time from a total of 15x10^6^ responder PBMCs. Additional file 3: Figure S2. Cytotoxic potential of HA-1-specific cells in late REP culture. Cells from REP D21 cultures were harvested and submitted to intracellular staining for flow cytometry analysis of CD107a and granzyme B production after restimulation with HA-1 peptide, with an irrelevant HLA-A0201-related peptide (LMP2 426-434) or with PMA/ionomycin; upper panels: representative plots and bottom panel: *n* = 4, data represent average ± SEM. Additional file 4: Figure S3. Generation of LMP2-specific T-cell lines in culture. **(A)** fold increased in the coculture step; *n* = 3. **(B)** fold increase in the rapid expansion protocol step; *n* = 3, data represent average ± SEM. **(C)** Percentage of CD8^+^ LMP2_426-434_^+^ cells generated over time in both culture steps analyzed by flow cytometry; *n* = 3, data represented as average ± SEM.

## Abbreviations

HSCT: Hematopoietic stem cell transplantation
APC: Antigen presenting cell
DC: Dendritic cell
EBV: Epstein-Barr virus
ELISpot: Enzyme-linked immunospot assay
GM-CFS: Granulocyte-macrophage colony-stimulating factor
GVHD: Graft-versus-host disease
GVL: Graft-versus-leukemia
HLA: Human leukocyte antigen
IFN: Interferon
IL: Interleukin
KLRG-1: Killer cell lectin-like receptor G1
LMP2: Latent membrane protein 2
MiHA: Minor histocompatibility antigen
NO: Nitric oxide
PBMCs: Peripheral blood mononuclear cells
PD-1: Programmed cell death-1
PE: R-phycoerythrin
PFA: Paraformaldehyde
PHA: Phytohemaglutinnin
PMA: Phorbol 12-myristate 13-acetate
PSG: Penicillin-Streptomycin-Glutamine
REP: Rapid expansion protocol
TIL: Tumor-infiltrating lymphocytes
TCR: T-cell receptor
TNF: Tumor necrosis factor
