# Supplementary figures and images for: Defining novel parameters for the optimal priming and expansion of minor histocompatibility antigen-specific T cells in culture
Source: J Transl Med. 2015 Apr 19;13:123. doi: 10.1186/s12967-015-0495-z (PMC4413989; doi:10.1186/s12967-015-0495-z)

**A**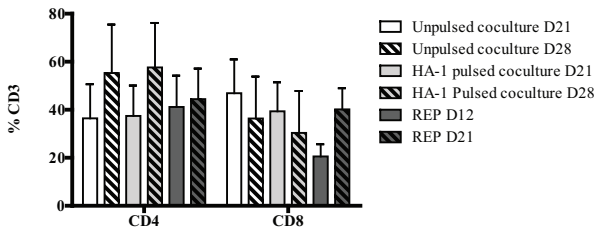**B**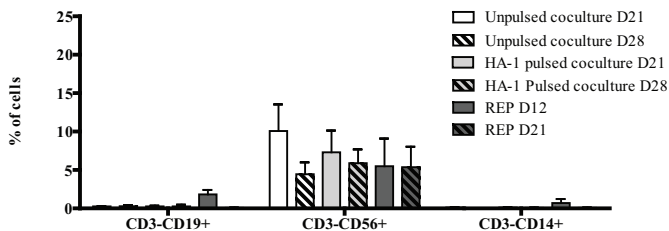**C**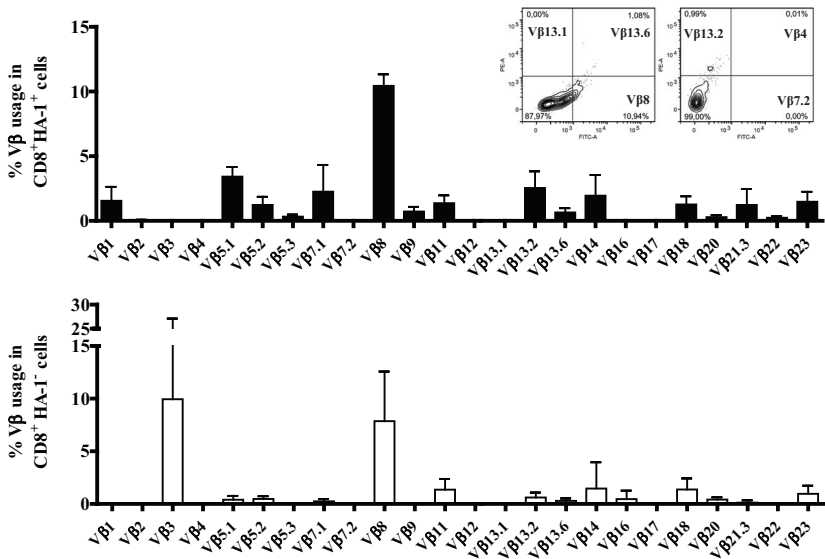

Supplement: Additional file 1: Figure S1. — Cell subpopulations and characteristics throughout cultures steps. (A) CD4+ and CD8+ T cells proportions analyzed by flow cytometry; n = 4, data show average ± SEM. (B) Percentage of contaminant such as B cells, NK cells and monocytes/macrophages analyzed by flow cytometry; n = 4, data represented as average ± SEM. (C) Clonogram representation (with representative examples in top right corner) of formerly identified TCR Vβ repertoire in CD8+ HA-1+ (upper panel) and CD8+ HA-1- (lower panel) cells at day 12 of the REP; n = 3, averages ± SEM. [file 12967_2015_495_MOESM1_ESM.pdf]

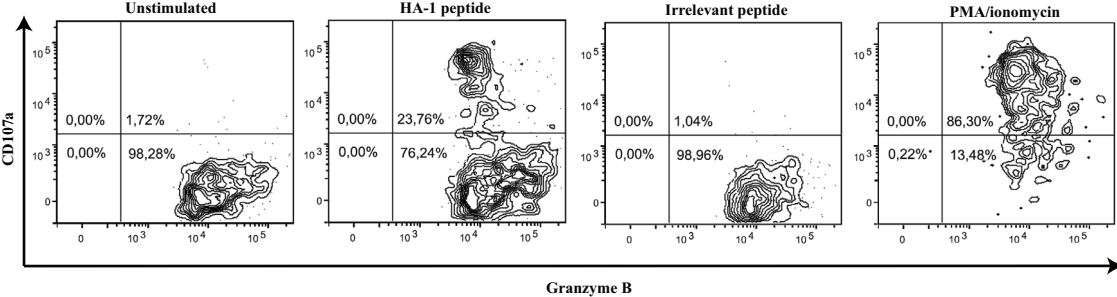

### REP D21

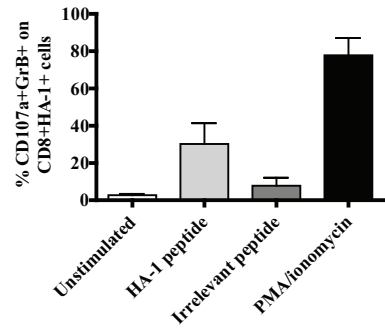

Supplementary Figure S2

Supplement: Additional file 3: Figure S2. — Cytotoxic potential of HA-1-specific cells in late REP culture. Cells from REP D21 cultures were harvested and submitted to intracellular staining for flow cytometry analysis of CD107a and granzyme B production after restimulation with HA-1 peptide, with an irrelevant HLA-A0201-related peptide (LMP2 426-434) or with PMA/ionomycin; upper panels: representative plots and bottom panel: n = 4, data represent average ± SEM. [file 12967_2015_495_MOESM3_ESM.pdf]

A

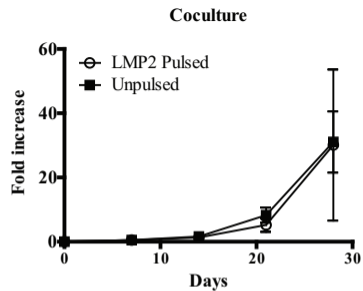

B

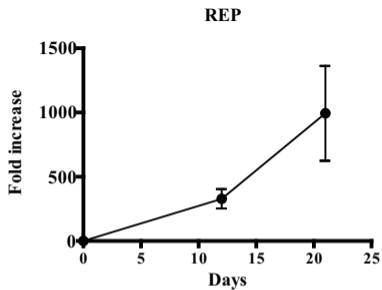

C

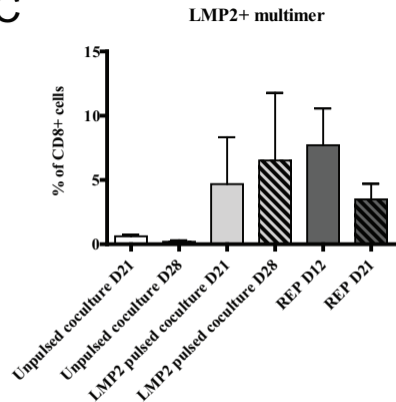

Supplementary Figure S3

Supplement: Additional file 4: Figure S3. — Generation of LMP2-specific T-cell lines in culture. (A) fold increased in the coculture step; n = 3. (B) fold increase in the rapid expansion protocol step; n = 3, data represent average ± SEM. (C) Percentage of CD8+ LMP2426-434 + cells generated over time in both culture steps analyzed by flow cytometry; n = 3, data represented as average ± SEM. [file 12967_2015_495_MOESM4_ESM.pdf]
